# CT-Based Radiomics Can Predict the Efficacy of Anlotinib in Advanced Non-Small-Cell Lung Cancer

**DOI:** 10.1155/2022/4182540

**Published:** 2022-12-26

**Authors:** Jingyu Chen, Chuhuai Wang, Weinuo Qu, Fangfang Liu, Zilin Zhou, Jiali Li, Qiongjie Hu, Qingguo Xie, Jinlin Wang, Qian Chu

**Affiliations:** ^1^Department of Oncology, Tongji Hospital, Tongji Medical College, Huazhong University of Science and Technology, Wuhan 430030, Hubei, China; ^2^Wuhan National Laboratory for Optoelectronics, Wuhan 430074, Hubei, China; ^3^Department of Radiology, Tongji Hospital, Tongji Medical College, Huazhong University of Science and Technology, Wuhan 430030, Hubei, China; ^4^Department of Biomedical Engineering, Huazhong University of Science and Technology, Wuhan 430074, Hubei, China

## Abstract

Anlotinib is a small-molecule RTK inhibitor that has achieved certain results in further-line treatment, but many patients do not respond to this drug and lack effective methods for identification. Although radiomics has been widely used in lung cancer, very few studies have been conducted in the field of antiangiogenic drugs. This study aims to develop a new model to predict the efficacy of patients receiving anlotinib by combining pretreatment computed tomography (CT) radiomic characters with clinical characters, in order to assist precision medicine of pulmonary cancer. 254 patients from seven institutions were involved in the study. Lesions were selected according to the RECIST 1.1 criteria, and the corresponding radiomic features were obtained. We constructed prediction models based on clinical, NCE-CT, and CE-CT radiomic features, respectively, and evaluated the prediction performance of the models for training sets, internal validation sets, and external validation sets. In the RAD score only model, the area under curve(AUC) of the NCE-CT cohort was 0.740 (95% CI: 0.622, 0.857) for the training set, 0.711 (95% CI: 0.480, 0.942) for the internal validation set, and 0.633(95% CI: 0.479, 0.787) for the external validation set, while that of the CE-CT cohort was 0.815 (95% CI: 0.705, 0.926) for the training set, 0.771 (95% CI: 0.539, 1.000) for the internal validation set, and 0.701 (95% CI: 0.489, 0.913) for the external validation set. In the RAD score-combined model, the AUC of the NCE-CT cohort was 0.796 (95% CI: 0.691, 0.901) for the training set, 0.579 (95% CI: 0.309, 0.848) for the internal validation set, and 0.590 (95% CI: 0.427, 0.753) for the external validation set, while that of the CE-CT cohort was 0.902 (95% CI: 0.828, 0.977) for the training set, 0.865 (95% CI: 0.696, 1.000) for the internal validation set, and 0.837 (95% CI: 0.682, 0.992) for the external validation set. In conclusion, radiomics has accurate predictions for the efficacy of anlotinib. CE-CT-based radiomic models have the best predictive potential in predicting the efficacy of anlotinib, and model predictions become better when they are combined with clinical characteristics.

## 1. Introduction

Lung cancer is the malignant tumor with the highest mortality rate in the world. In 2020, the incidence rate of lung carcinoma was 11.4% and the mortality rate was as high as 18% [1]. With the advent of targeted therapy and immunotherapy, treatment options for lung cancer patients have become more diverse, and the overall survival also continues to prolong. The 3-year survival rate rose from 19% in 2001 to 31% in 2015 through 2017, and the median survival increased from 8 to 13 months, with the five-year survival rate for non-small-cell lung cancer (NSCLC) being higher than that for small cell lung cancer [2, 3]. As treatment options become more available, how to make a patient's treatment decision remains controversial.

Receptor tyrosine kinases (RTKs) are single-pass membrane proteins closely related to cell growth, motility, differentiation, and survival. They are grouped into subfamilies based on the similarity of their extracellular domains, including vascular endothelial growth factor (VEGF) or fibroblast growth factor receptor (FGFR) families [4]. Types of tyrosine kinase inhibitors (TKIs) demonstrate effects on inhibition of angiogenesis; however, only a few TKIs, including nintedanib and anlotinib, have shown positive anticancer effects [5]. Anlotinib is a small-molecule RTK inhibitor that targets VEGFR1, VEGFR2/KDR, VEGFR3, and Raf serine/threonine kinases, platelet-derived growth factor receptor PDGFR-*α*, and FGFR1, FGFR2, and FGFR3 [6, 7]. Therefore, anlotinib can inhibit angiogenesis in tumors and limit tumor growth. ALTER-0303 was a randomized, double-blind, placebo-controlled, multicenter, phase III trial that compared the efficacy and safety of anlotinib with that of the placebo in patients with advanced NSCLC who progressed after at least two lines of prior treatments. The results showed that anlotinib was more effective than the placebo in third-line treatment in patients with advanced NSCLC [8]. Though anlotinib has achieved certain results in further-line treatment, there are still many patients who do not respond to this drug. How to filtrate patients effectively for anlotinib remains unclear.

Different from traditional needle biopsy methods to obtain local tissue samples, radiographic images can fully display the overall characteristics of tumors to analyze the heterogeneity of tumors. Based on machine learning, radiomics has been widely used in the diagnosis and treatment of oncology. At present, many studies have used radiomics to detect the pathological type, gene mutation, programmed death ligand 1 (PD-L1) expression level, or tumor-infiltrating lymphocyte (TILs) levels in patients and even predict the efficacy of types of treatment [9–15]. A meta-analysis showed that the pooled diagnostic odds ratio for predicting immunotherapy response in NSCLC using radiomics was 14.99 (95% CI: 8.66–25.95) [16]. These research studies demonstrate the potential of radiomics for solving clinical problems.

Although radiomics has been widely used in lung cancer, very few studies have been conducted in the field of antiangiogenic drugs. This study aims to develop a new model to predict the efficacy of patients receiving anlotinib by combining pretreatment computed tomography (CT) radiomic characters with clinical characters, in order to assist precision medicine of pulmonary cancer. In this study, patients who underwent noncontrast-enhanced CT (NCE-CT) and CE-CT were divided into two groups for feature extraction and training separately [17].

## 2. Materials and Methods

### 2.1. Patient Population

The data on the training set and the internal validation set was retrieved from a clinical trial: “A Real-world Study: Efficacy and Safety of Anlotinib for Advanced Non-small Cell Lung Cancer (NSCLC)” (NCT04871997). The data on the external set was obtained from the medical record system of Tongji Hospital, Tongji Medical College, Huazhong University of Science and Technology, and these patients did not participate in the clinical trial. In this retrospective study, all the patients had been getting recruited since July 2019, ending in September 2022. Data had been getting analyzed since October 2022. This study was approved by the Institutional Ethics Committee of Tongji Medical College, Huazhong University of Science and Technology (approval number S1040). Informed consent was obtained from all patients.

  Inclusion criteria were as follows: (1) age: ≥18 years old, no gender limit; (2) diagnosed as advanced non-small-cell lung cancer; (3) at least one tumor lesion in the lung was not subjected to local treatment such as irradiation in the past and could be accurately measured, and the longest diameter was ≥10 mm; (4) the patient received two cycles of medication and at least had efficacy assessment performed once. (5) Patients included in the training set needed to achieve the median progression-free survival (PFS). If any of the above items was “no,” the patient was not suitable for this study.
  Exclusion criteria were as follows: (1) those who were confirmed to be allergic to anlotinib and/or its excipients; (2) patients with anlotinib contraindications. Finally, 254 patients were retrospectively involved in this study.

### 2.2. CT Imaging Protocol

As shown in Table S1, all CT images were acquired by using a multislice CT system, with a tube voltage of 120 kVp and automatic tube current modulation. Each CT image was reconstructed with an image matrix of 512 × 512 pixels, while the slice thickness of CT scans was 1.0 to 2.0 mm with coverage from the apex to the bottom of the lungs. Images were saved in the DICOM format.

### 2.3. Region-of-Interest Segmentation and Feature Extraction

Tumor lessons were segmented by a chest radiology specialist with 1 year of experience using open-source software, 3D-slicer (version 4.11, http://www.slicer.org). A senior imaging physician (LJL with 7 years of experience) performed the identification of region-of-interest (ROI) regions. Intrapulmonary lesions were selected as per the RECIST version 1.1 criteria [18], and for patients possessing multiple evaluable lesions in the lungs, the five lesions with the largest diameter were selected for outlining. Using the “threshold” semiautomatic segmentation tool in the range of WW −500∼−100 HU and WL 200–400 HU to segment the lesions in the lungs confirmed the extent of the tumor and excluded vascular and gas shadows within the tumor. The average radiomic features of all target lesions were used as global radiomic features to predict treatment response.

A total of 851 original radiomic features were extracted using a PyRadiomics (version 3.0.1: Computational Imaging and Bioinformatics Lab, Harvard Medical School) module in 3D-slicer from these ROIs, consisting of first-order features (*N* = 18), shape features (*N* = 14), and texture features (*N* = 75) extracted from preprocessed original images, and wavelet features (*N* = 744) were extracted from processed images with wavelet filters. The category of features is displayed in Table S2.

### 2.4. Radiomic Feature Selection and Radiomic Signature Development

This study complied with the IBSI guidelines in general. To avoid the information loss of processing, we did not perform any special pretreatment for radiographic imaging considering that the difference in CT parameters in this study were not particularly large [19]. After obtaining image omics parameters, the *t*-test was conducted for each omics parameter based on the corresponding prognosis. Parameters with *P* < 0.1 were screened for subsequent LASSO logistic modeling. LASSO logistic regression was used for dimension reduction and identifying the most PFS-related features from repeatable and nonredundant features in the training set. Patients of NCE-CT and CE-CT were randomly divided into training and validation sets at the ratio of 3 : 1. The radiomic score (RAD score) was calculated through a linear combination of selected features weighted by their respective coefficients.

### 2.5. Outcome Measures

#### 2.5.1. Primary Outcome Measures

*(1) Progression-Free Survival (PFS)*. It is the length of time during and after the treatment of a disease, such as cancer, that a patient lives with the disease, but it does not get worse.

#### 2.5.2. Secondary Outcome Measures

*(1) Overall Survival (OS)*. It is the length of time from either the date of diagnosis or the start of treatment for a disease, such as cancer, that patients diagnosed with the disease are still alive.

*(2) Objective Response Rate (ORR)*. It is the percentage of patients on whom a therapy has some defined effect; for example, cancer shrinks or disappears after treatment.

*(3) Disease Control Rate (DCR)*. It is the percentage of patients with advanced or metastatic cancer who have achieved complete response, partial response, and stable disease with a therapeutic intervention in clinical trials of anticancer agents.

### 2.6. Statistical Analysis

Statistical analyses were performed using IBM SPSS Statistics (version 25.0, https://www.ibm.com), GraphPad Prism (version 9.3.1, https://www.graphpad.com), R software (version 4.0.5, https://www.r-project.org), and Python (version 3.9, https://www.python.org). Kaplan–Meier analysis and Cox proportional hazard regression were performed using GraphPad Prism. The *χ*2 test and hazard regression were performed using IBM SPSS Statistics. The glmnet R package was used for the LASSO regression method. The rms, pROC, lattice, ggplot2, Hmisc, and rmda R packages were used to plot the nomogram, receiver operating characteristic (ROC) curves, and decision curve analysis (DCA) wherever appropriate. The calibration curves were plotted by using Python packages named Matplotlib.

## 3. Results

### 3.1. Patient Population

**Figure 1:**
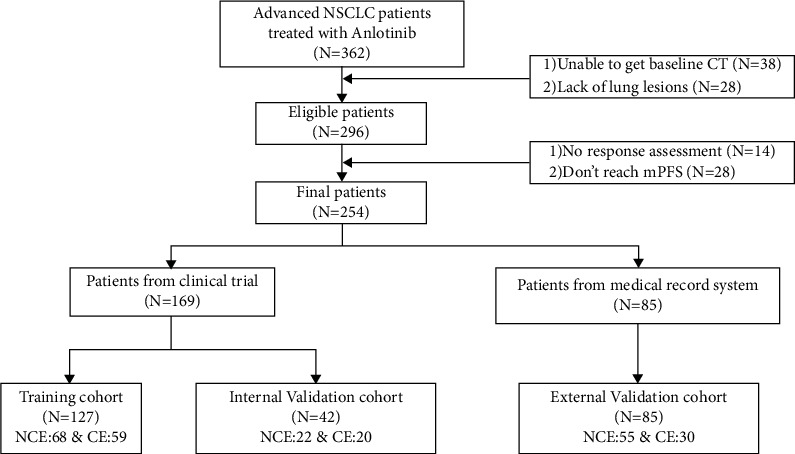
The participants' flow-chart of this study.

In this study, complete treatment history and follow-up data were collected from 362 patients, and after censoring 38 patients who were unable to obtain baseline CT, 28 patients who lacked intrapulmonary lesions, and 14 patients who did not obtain imaging efficacy assessment, 282 patients were included in the study. CT was performed within 14 days prior to drug administration. Patients included in the model training set were required to achieve the median PFS or disease progression, so 28 patients were excluded. Finally, 254 patients were included in the study. The participants' flowchart is displayed in Figure 1.

Baseline patient characteristics are summarized in Table 1. There was no significant difference in the training set, the internal validation set, and the external validation set in terms of sex, age, smoking, Eastern Cooperative Oncology Group Performance Status (ECOG PS) score, metastasis lessons, pathological type, treatment line, and best response.

For the training set and internal validation set, the overall ORR and DCR were 21.2% and 85.9%, respectively. The median PFS was 6.4 (95% CI: 5.7, 7.1) months, and the median OS was 12.9 (95% CI: 5.8, 20.0) months. For the external validation set, the ORR and DCR were 18.8% and 87.1%, respectively. The median PFS was 5.1 95% CI: 3.2, 7.1 months, and the median OS was 14.4 (95% CI: 8.2, 20.6) months.

### 3.2. Acquisition of Radiomic Features and Construction of Models

**Figure 2:**
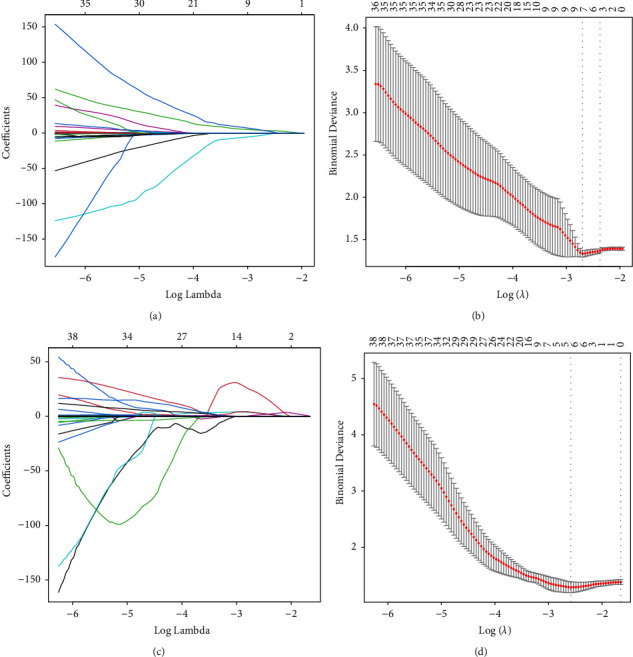
LASSO regression process of the NCE-CT cohort (a, b) and the CE-CT cohort (c, d).

A total of 851 radiomic features were extracted from CT images for each patient. These extracted features were preprocessed using *t*-tests to remove redundant and irrelevant features. Then, LASSO regression modified 7 features for the NCE-CT cohort and 5 features for the CE-CT cohort, which were mostly associated with PFS in the training set. The RAD score was calculated through a linear combination of selected features weighted by their respective coefficients (Appendix S1). The training process is shown in Figure 2.

It can be observed that NCE-CT contains one parameter related to tumor size and that all other parameters are texture features, while the CE-CT parameters are all texture features. CE-CT is able to better visualize microvascular changes within the tumor due to the presence of contrast agents, thus enabling a better characterization of tumor heterogeneity, which in turn has better potential for predicting the efficacy of anlotinib.

### 3.3. Predictive Validity of the Model

**Figure 3:**
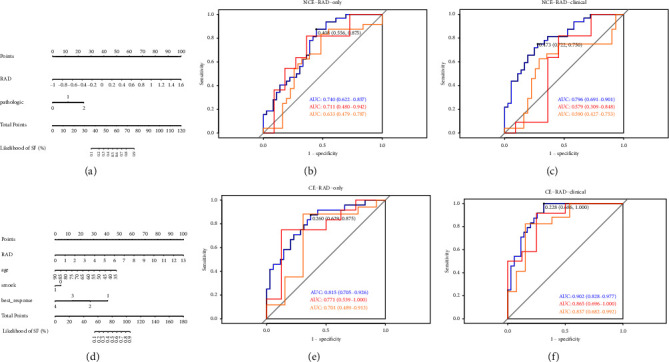
Nomogram plot and the ROC curve of the NCE-CT cohort and the CE-CT cohort. (a, d) The nomogram plot for the NCE-CT and CE-CT cohort; (b, e) the ROC curve of the model based on the RAD score only of the NCE-CT and CE-CT cohort; (c, f) the ROC curve of the model based on the RAD score combined with clinical characteristics of the NCE-CT and CE-CT cohort.

After performing logistic regression for the NCE-CT cohort and the CE-CT cohort separately, the RAD score was used to set up a model in conjunction with the logistic regression results, and we also performed modeling using the RAD score alone to obtain a total of four ROC charts and two nomogram plots (Figure 3). In the RAD score only model, the area under curve(AUC) of the NCE-CT cohort was 0.740 (95% CI: 0.622, 0.857) for the training set, 0.711 (95% CI: 0.480, 0.942) for the internal validation set, and 0.633(95% CI: 0.479, 0.787) for the external validation set, while that of the CE-CT cohort was 0.815 (95% CI: 0.705, 0.926) for the training set, 0.771 (95% CI: 0.539, 1.000) for the internal validation set, and 0.701 (95% CI: 0.489, 0.913) for the external validation set. In the RAD score-combined model, the AUC of the NCE-CT cohort was 0.796 (95% CI: 0.691, 0.901) for the training set, 0.579 (95% CI: 0.309, 0.848) for the internal validation set, and 0.590 (95% CI: 0.427, 0.753) for the external validation set, while that of the CE-CT cohort was 0.902 (95% CI: 0.828, 0.977) for the training set, 0.865 (95% CI: 0.696, 1.000) for the internal validation set, and 0.837 (95% CI: 0.682, 0.992) for the external validation set. It can be clearly seen that for the model with the RAD score alone, the NCE cohort exhibited less than optimal predictive validity, while the CE cohort showed some predictive power. After adding clinical factors to the model, the predictive power of the NCE cohort decreased, while the predictive power of the CE cohort significantly improved.

**Figure 4:**
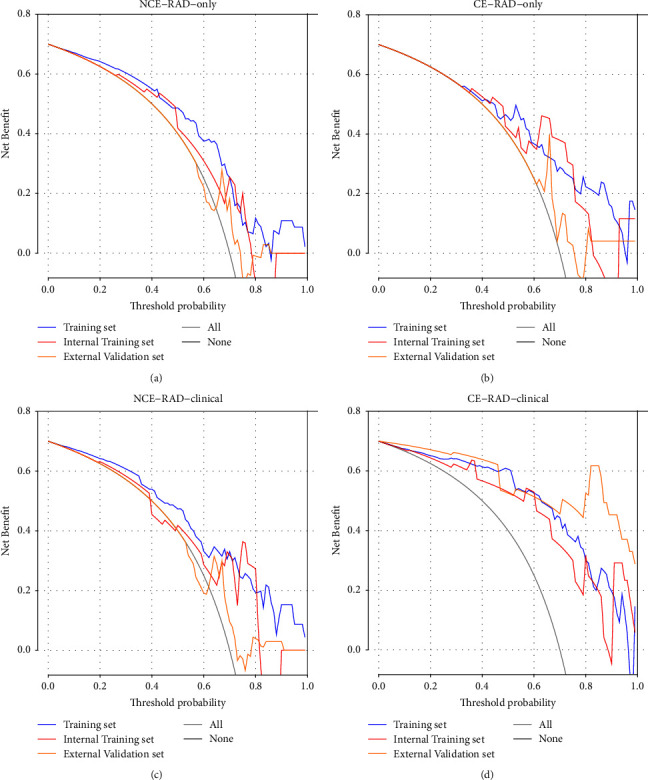
DCA of the training set and the internal and external validation set of four models based on the RAD score only and a combination of radiomics and clinical features, respectively. (a) NCE radiomic only model; (b) CE radiomic only model; (c) NCE radiomic-combined model; (d) CE radiomic-combined model.

DCA was performed for the above two radiomic only models as well as the model built based on radiomics and clinical features. As shown in Figure 4, the training sets of all four models have good DCA fits, while the validation sets of the combination of the radiomic and clinical feature model and the CE-CT radiomic only model have better DCA performance. The NCE-CT radiomic only model has relatively poor results, which also coincide with the results of the ROC curve.

### 3.4. Verification of the Predictive Power of the Model in the External Validation Set

**Figure 5:**
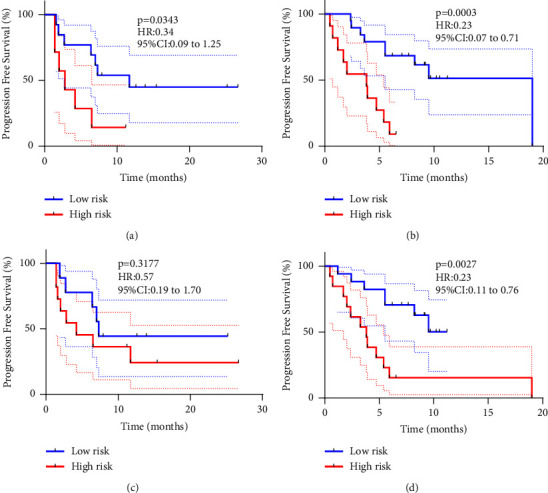
Kaplan–Meier survival analyses of PFS between the low- and high-risk groups in the CE-CT cohort based on the RAD score only and a combination of radiomics and clinical features, respectively. (a) The CE radiomic only model in the internal validation set; (b) the CE radiomic only model in the external validation set; (c) the CE radiomics-combined model in the internal validation set; (d) the CE radiomic-combined model in the external validation set.

The Youden index in the ROC curve was used as our threshold for differentiating patients into high-risk and low-risk populations (Figures 3(b) and 3(e)) represent the radiomic only models, and Figures 3(c) and 3(f) represent the radiomic-combined models). Patients with logistic scores below this threshold were in the high-risk group, and vice versa in the low-risk group, and the PFS showed a significant difference in the internal and external validation set. As shown in Figure 5, both the radiomic only model and the combination model have excellent PFS prediction ability for patients in the enhanced CT cohort, both in the internal validation set (Figures 5(a) and 5(c), *P*=0.0343; *P*=0.3177) and the external validation set (Figures 5(b) and 5(d), *P*=0.0003; *P*=0.0027).

**Figure 6:**
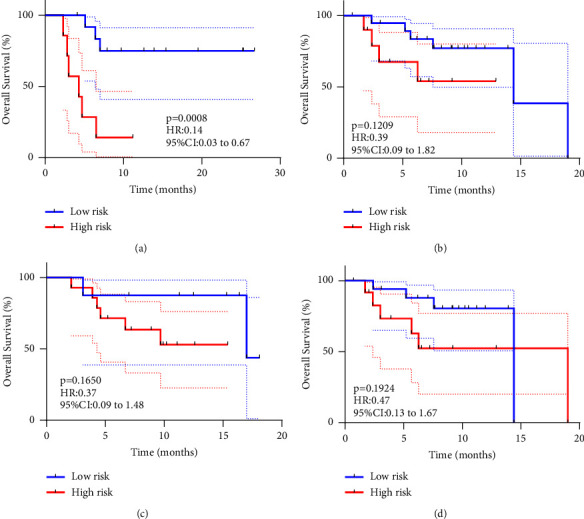
Kaplan–Meier survival analyses of OS between the low- and high-risk groups in the CE-CT cohort. (a) The CE radiomic only model in the internal validation set; (b) the CE radiomic only model in the external validation set; (c) the CE radiomic-combined model in the internal validation set; (d) the CE radiomic-combined model in the external validation set.

In terms of OS, low-risk patients were significantly higher than high-risk patients in the internal validation set of the radiomic only model (Figure 6(a), *P*=0.0008). For the external validation set, the radiomic only model and in both sets of the radiomic-combined model, the difference in OS was not significant between the high-risk and low-risk populations, but we can still see certain trends through the KM curves (Figures 6(b)–6(d)), *P*=0.1209; *P*=0.1650; *P*=0.1924). Despite the excellent predictive validity of the prediction model built on CE-CT, the predictive capability of the NCE-CT-based prediction model was not satisfactory for the prognosis of this cohort of patients. The NCE-CT-based model was unable to distinguish between the prognosis of the high-risk and low-risk cohorts for either PFS or OS (Figures S1 and S2).

## 4. Discussion

Radiomics has been widely used in the whole process of cancer treatment, among which the clinical application of tumor antiangiogenic drugs and imaging in lung cancer has been very extensive. Most of the studies on antitumor angiogenic drugs are focused on bevacizumab, and most of the tumor types are recurrent glioma and gastrointestinal tumors. Several studies have used MRI-based radiomics to predict the efficacy of bevacizumab in patients with recurrent glioma, and excellent predictive validity has been observed for PFS and OS [20, 21]. In gastrointestinal tumors, CE-CT radiomic studies of patients with colon and liver cancers were successful in predicting the efficacy of bevacizumab in patients receiving bevacizumab [22, 23].

Compared to the above studies, radiomic studies targeting small-molecule antitumor angiogenic agents are very rare. PET, CT, and MRI-based radiomics in kidney cancer can effectively predict early response and survival with sunitinib but mostly in small sample studies [24–26]. Another small sample study explored the efficacy of combined CT and methotrexate-based prediction of apatinib for advanced hepatocellular carcinoma with favorable results [27]. Despite the scarcity of such studies, they show us the good potential of radiomics for the prediction of efficacy of antitumor angiogenic drugs.

The study of predicting the efficacy of antitumor angiogenic drugs in lung cancer by radiomics is scarce. In our study, we built models to predict the efficacy of antiangiogenic drugs based on NCE-CT and CE-CT, respectively. The radiomic features incorporated into the modeling are predominantly second-order features, implying that texture features act as primary predictive correlates, and we found that the models constructed using CE-CT images had good predictive validity and were more effective when clinical factors were added. The NCE-CT-based model did not have such good predictive efficacy. There was one study using both CE-CT and NCE-CT images to model the predicted prognosis of immunotherapy, but there was no significant difference in the predictive performance of the two models [28]. In another study, using CE-CT and NCE-CT to predict EGFR mutation status in NSCLC patients, the predictive performance of the two methods also did not differ significantly [29]. Anlotinib acts as an antiangiogenic agent, which primarily affects the tumor microvasculature that has an effect on the tumor. Due to the presence of contrast agents, CE-CT is able to better visualize microvascular changes within the tumor. This may be the reason why the model based on CE-CT in our article was able to better predict the efficacy of anlotinib.

This article, as the first article exploring NCE-CT versus CE-CT in the prediction of anlotinib and with patients derived from clinical studies, has high credibility of information and bright results but still leaves some questions. (1) Heterogeneity of imaging parameters resulting from multicenter studies may affect study results because no resampling was performed. However, there is not much difference in parameters between individual CT instruments. We also performed an external validation and confirmed that our model has good stability and accuracy. (2) Heterogeneity between the training and validation sets might exist after grouping due to insufficient number of patients. (3) Anlotinib, as a drug developed in China, data are only available for the Chinese population. (4) This is a real-world study, and patients may be combined with other treatment options during the course of their treatment, which may affect the accuracy of the results.

## 5. Conclusions

Anlotinib has good efficacy in the treatment of advanced non-small-cell lung cancer. Radiomics has accurate predictions in the efficacy of anlotinib. CE-CT-based radiomic models have the best predictive potential in predicting the efficacy of anlotinib, and model predictions become better when they are combined with clinical characteristics.

## Figures and Tables

**Table 1:** Baseline characteristics of patients.

Characteristic	NCE-CT cohort				CE-CT cohort			
	Training set (*n* = 68)	Internal validation set (*n* = 22)	External validation set (*n* = 55)	*P* value	Training set (*n* = 59)	Internal validation set (*n* = 20)	External validation set (*n* = 30)	*P* value
Sex				0.826				0.001
Male	45	13	36		37	19	22	
Female	23	9	19		22	1	8	
Age				0.640				0.268
<65	36	11	33		32	8	19	
≥65	32	11	22		27	12	11	
Tobacco use				0.464				0.716
Smoker	29	7	26		29	11	13	
Nonsmoker	39	15	29		30	9	17	
ECOG PS				0.193				0.249
0∼1	56	19	39		47	15	19	
≥2	12	3	16		12	5	11	
Metastasis lessons ≥3				0.453				0.729
Yes	9	4	12		8	3	6	
No	59	18	43		51	17	24	
Brain metastases				0.789				0.781
Yes	6	3	5		7	3	5	
No	62	19	50		52	17	24	
Liver metastases				0.284				0.500
Yes	6	0	6		5	2	5	
No	62	22	49		54	18	25	
Bone metastases				0.699				0.729
Yes	13	5	14		8	3	6	
No	55	17	41		51	17	24	
Pathological type				0.055				0.615
Adenocarcinoma	46	12	44		40	11	21	
Squamous cell carcinoma	20	10	8		17	9	8	
Others	2	0	3		2	0	1	
Treatment line				0.090				0.137
1	6	5	5		3	0	0	
2	14	4	20		8	5	10	
3	48	13	30		48	15	20	
Best response				0.283				0.432
PR	19	5	8		12	2	8	
SD	40	14	38		35	14	20	
PD	5	3	9		10	4	2	

## Data Availability

The raw data and source codes used to support the findings of this study have been deposited in the GitHub repository (https://github.com/ChuhuaiWang/data_analysis_code).

## Supplementary Materials

Supplementary Materials Supplement data are shown in Supplementary Materials. The file contains the CT scanning protocol (Table S1), the category of radiomic features (Table S2), and the formula for the radiomic score (Appendix S1). Kaplan–Meier curves of the NCE-CT cohort are shown in Figure S1 and S2.
