# Supplementary material for: CT-Based Radiomics Can Predict the Efficacy of Anlotinib in Advanced Non-Small-Cell Lung Cancer
Source: J Oncol. 2022 Dec 26;2022:4182540. doi: 10.1155/2022/4182540 (PMC9807313; doi:10.1155/2022/4182540)
Supplement: Supplementary Materials — Supplement data are shown in Supplementary Materials. The file contains the CT scanning protocol (Table S1), the category of radiomic features (Table S2), and the formula for the radiomic score (Appendix S1). Kaplan–Meier curves of the NCE-CT cohort are shown in Figure S1 and S2. [file 4182540.f1.docx]

Table S1: CT scanning protocol

| Institution | Manufacturer | Manufacturer’s Model Name | Tube Voltage | Matrix | Thickness |
| --- | --- | --- | --- | --- | --- |
| Enshi Prefecture Central HP | Philips | Ingenuity CT | 120 kVp | 512*512 | 1 mm |
| Xiangyang Central Hosp. | Philips | Ingenuity CT | 120 kVp | 512*512 | 2 mm |
| XiangYang NO.1 Hospital | Siemens | SOMATOM go.top | 120 kVp | 512*512 | 1 mm |
| Wuhan TongJi Hospital | GE MEDICAL SYSTEM | LightSpeed 16 | 120 kVp | 512*512 | 1.25 mm |
| Shiyan People Hospital | Siemens | SOMATOM Definition AS+ | 120 kVp | 512*512 | 1 mm |
| Hubei Xiaogan Center Hospital | Philips | Ingenuity CT | 120 kVp | 512*512 | 1 mm |
| Yichang Central Hospital | Siemens | Brilliance 16 | 120 kVp | 512*512 | 2 mm |

Table S2: Feature Category

| Feature Category | Feature Numbers | Feature Name |
| --- | --- | --- |
| Shape-related Statistics | 14 | Elongation  Flatness  Least Axis Length  Major Axis Length  Maximum 2D Diameter (Column)  Maximum 2D Diameter (Row)  Maximum 2D Diameter (Slice)  Maximum 3D Diameter  Mesh Volume  Minor Axis Length  Sphericity  Surface Area  Surface Volume Ratio  Voxel Volume |
| First Order Histogram Features | 18 | 10 Percentile  90 Percentile  Energy  Entropy  Interquartile Range  Kurtosis  Maximum  Mean Absolute Deviation  Mean  Median  Minimum  Range  Robust Mean Absolute Deviation  Root Mean Squared  Skewness  Total Energy  Uniformity  Variance |
| Gray Level Co-occurrence Matrix (GLCM) | 24 | Autocorrelation  Cluster Prominence  Cluster Shade  Cluster Tendency  Contrast  Correlation  Difference Average  Difference Entropy  Difference Variance  ID (inverse difference)  IDM (inverse difference moment)  IDMN (inverse difference moment normalized)  IDN (inverse difference normalized)  IMC1 (Informational measure of correlation 1)  IMC2 (Informational measure of correlation 2)  Inverse Variance  Joint Average  Joint Energy  Joint Entropy  MCC  Maximum Probability  Sum Average  Sum Entropy  Sum Squares |
| Gray Level Dependence Matrix (GLDM) | 14 | Dependence Entropy  Dependence Non-Uniformity  Dependence Non-Uniformity Normalized  Dependence Variance  Gray Level Non-Uniformity  Gray Level Variance  High Gray Level Emphasis  Large Dependence Emphasis  Large Dependence High Gray Level Emphasis  Large Dependence Low Gray Level Emphasis  Low Gray Level Emphasis  Small Dependence Emphasis  Small Dependence High Gray Level Emphasis  Small Dependence Low Gray Level Emphasis |
| Gray Level Run Length Matrix (GLRLM) | 16 | Gray Level Non-Uniformity  Gray Level Non-Uniformity Normalized  Gray Level Variance  High Gray Level Run Emphasis  Long Run Emphasis  Long Run High Gray Level Emphasis  Long Run Low Gray Level Emphasis  Low Gray Level Run Emphasis  Run Entropy  Run Length Non-Uniformity  Run Length Non-Uniformity Normalized  Run Percentage  Run Variance  Short Run Emphasis  Short Run High Gray Level Emphasis  Short Run Low Gray Level Emphasis |
| Gray Level Size Zone Matrix (GLSZM) | 16 | Gray Level Non-Uniformity  Gray Level Non-Uniformity Normalized  Gray Level Variance  High Gray Level Zone Emphasis  Large Area Emphasis  Large Area High Gray Level Emphasis  Large Area Low Gray Level Emphasis  Low Gray Level Zone Emphasis  Size Zone Non-Uniformity  Size Zone Non-Uniformity Normalized  Small Area Emphasis  Small Area High Gray Level Emphasis  Small Area Low Gray Level Emphasis  Zone Entropy  Zone Percentage  Zone Variance |
| Neighborhood Gray Tone Difference Matrix (NGTDM) | 5 | Busyness  Coarseness  Complexity  Contrast  Strength |
| Wavelet transform | 744 | Wavelet features |

Appendix S1: Equations of Radiomics Score

Rad-score_NCE_ = 1.3398590340 × wavelet_HHH_glcm_MCC

+ 0.4308022893 × wavelet_HLL_glcm_MCC

- 0.0008763161 × original_shape_Maximum2DDiameterRow

- 0.0061217412 × wavelet_LHH_gldm_LargeDependenceLowGrayLevelEmphasis

- 0.1270287208 × wavelet_LLL_glrlm_RunLengthNonUniformityNormalized

- 2.7352452113 × wavelet_LLH_glszm_GrayLevelNonUniformityNormalized

- 4.7681701925 × wavelet_HHL_glcm_Imc1

Rad-score_CE_ = 20.3962048*wavelet-LHL-ngtdm-Contrast

+2.7712365* wavelet-LHH-glcm-Imc2

+32.7640920* wavelet-HHL-ngtdm-Strength

+1.2483906* wavelet-HHH-glcm-Imc2

+0.0534004* wavelet-LLL-glrlm-RunVariance

Figure S1: Kaplan–Meier survival analyses of PFS between the low and high-risk groups in NCE-CT cohort.


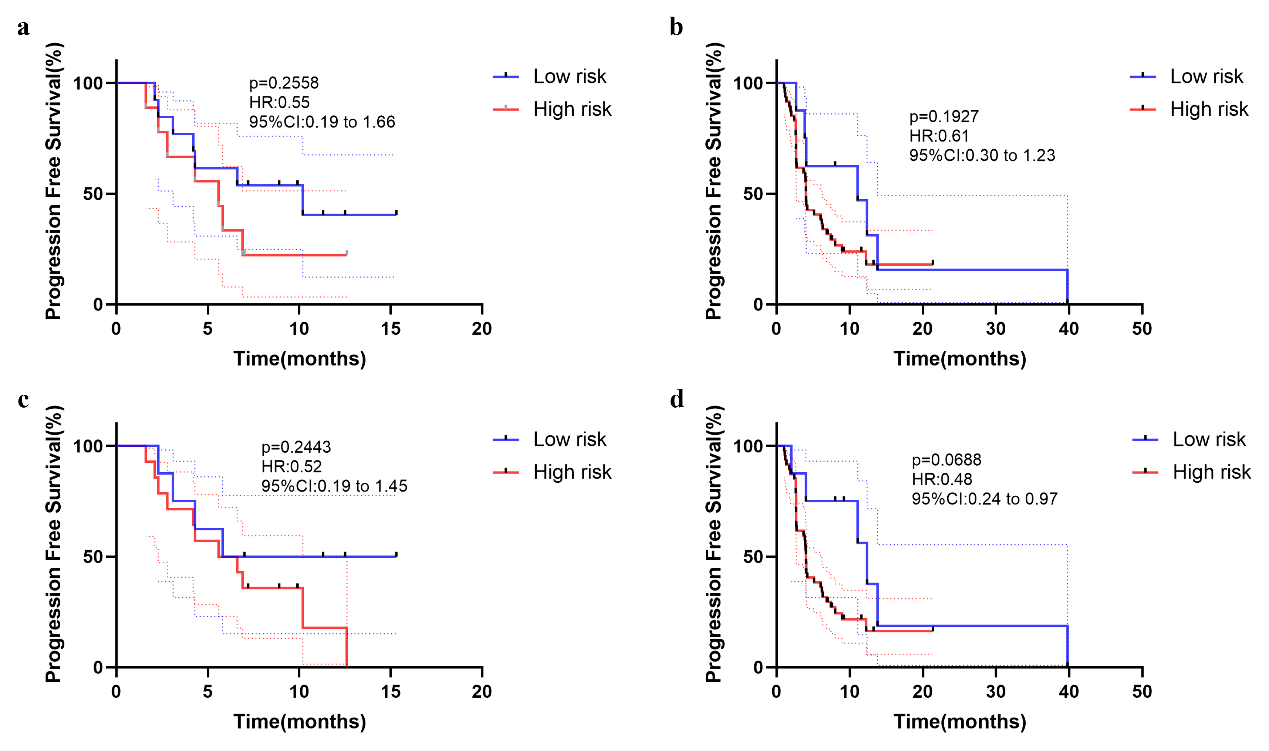


a: NCE radiomics-only model in internal set;

b: NCE radiomics-only model in external set;

c: NCE radiomics-combined model in internal set;

d: NCE radiomics-combined model in external set.

Figure S2: Kaplan–Meier survival analyses of OS between the low and high-risk groups in NCE-CT cohort.


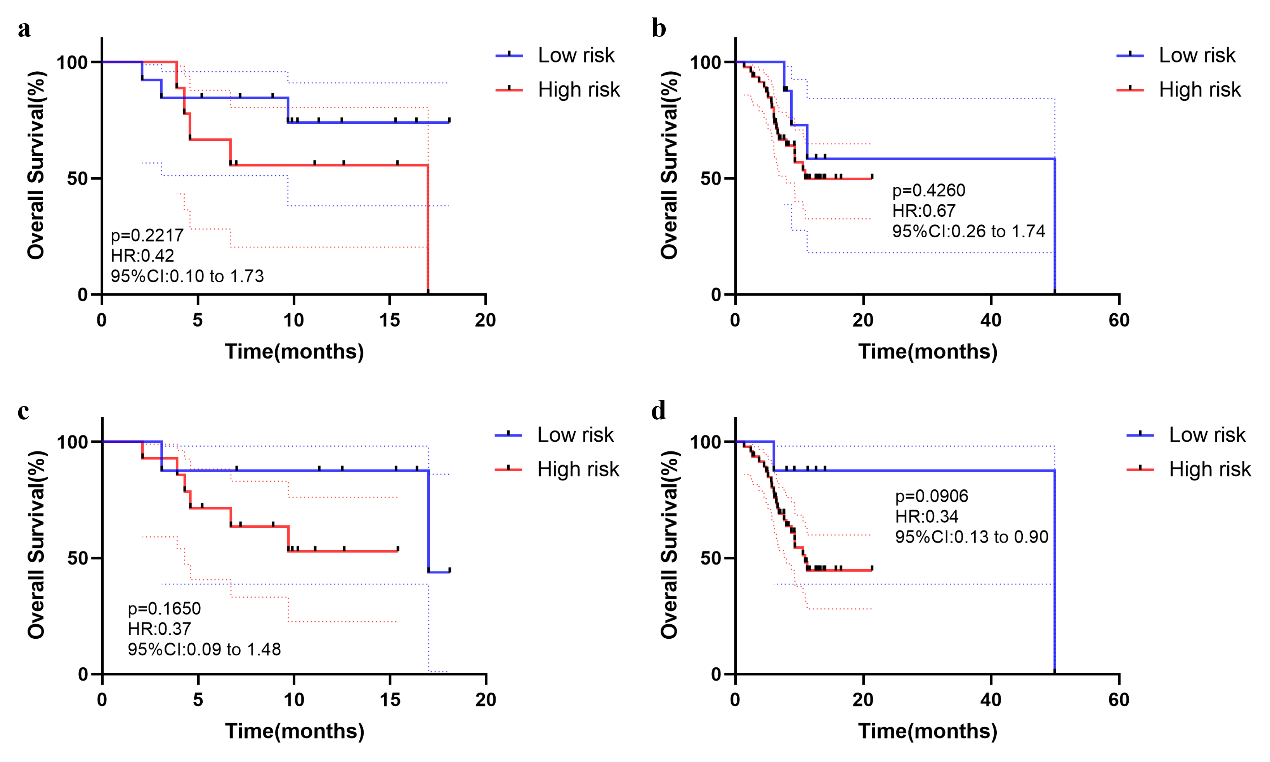


a: NCE radiomics-only model in internal set;

b: NCE radiomics-only model in external set;

c: NCE radiomics-combined model in internal set;

d: NCE radiomics-combined model in external set.
